# A case of a warfarinized renal cancer patient monitored for prothrombin time-international normalized ratio during methadone introduction

**DOI:** 10.1186/s40981-017-0092-7

**Published:** 2017-06-12

**Authors:** Kaoru Yoshioka, Katsuya Ohmori, Soshi Iwasaki, Kazunobu Takahashi, Akemi Sato, Hiromasa Nakata, Atsushi Miyamoto, Michiaki Yamakage

**Affiliations:** 1 Division of Hospital Pharmacy, Sapporo Medical University, South1, West16, Chuo-ku, Sapporo-city, Hokkaido Japan; 2 Department of Nursing, Sapporo Medical University, South1, West16, Chuo-ku, Sapporo-city, Hokkaido Japan; 3 Department of Anesthesiology, Sapporo Medical University, South1, West16, Chuo-ku, Sapporo-city, Hokkaido Japan; 4 Ain Holdings and Nitori Holdings Department of Palliative Medicine, Sapporo Medical University, South1, West16, Chuo-ku, Sapporo-city, Hokkaido Japan

**Keywords:** Methadone, Warfarin, CYP2C9, CYP3A4, CYP2D6, CYP2B6, Prothrombin time-international normalized ratio, Drug interaction

## Abstract

**Background:**

Warfarin, a widely used anticoagulant, interacts with various agents used in palliative care, such as oxycodone, morphine, acetaminophen, and non-steroidal anti-inflammatory drugs (NSAIDs); however, there are no reports of its interaction with methadone. We report a case of a patient receiving warfarin when methadone was introduced for pain control with monitoring of the prothrombin time-international normalized ratio (PT-INR) and deduced the pharmacological background.

**Case presentation:**

A 60-year-old male was emergently admitted to our university hospital for the sudden onset of severe back pain. Abdominal CT imaging revealed that the vertebral body of the ninth thoracic vertebra was occupied by bone metastasis and crushed, which caused his back pain. He received warfarin 3.5 mg/day for atrial fibrillation and tapentadol 100 mg p.o. daily for pain relief. The prothrombin time-international normalized ratio (PT-INR) was maintained at >2.2. The patient’s history included diabetes mellitus and hypertension, but his laboratory test was unremarkable with the exception that his eGFR was 34 ml/min.

Initially, a fentanyl dermal patch was used instead of tapentadol to avoid interactions with warfarin. We started concomitant administration of oxycodone and 2.4 g/day of acetaminophen while monitoring the PT-INR because acetaminophen increased the PT-INR to 2.93. A continuous intravenous infusion of oxycodone was introduced, in increments of the dose, resulting in an increase of the PT-INR to 3.41, which is required to reduce the dose of warfarin to 1.5 mg. Because of the lack of effective pain relief, methadone was introduced and the dose was gradually increased. The PT-INR was not changed and the dose of warfarin was not changed. An infusion of oxycodone and oral methadone was used to allow the patient to walk in his room, and he was later transferred to the palliative hospital.

**Conclusions:**

In an oral warfarinized patient, methadone seemed to undergo different metabolism than oxycodone. When warfarin and methadone are used together, we have to consider their interaction by comparing the competitive inhibition of CYP2C9 to the induction of CYP3A4 by methadone, because CYP3A4 metabolize various drugs including oxycodone.

## Background

Cancer patients are often affected by complications and require multiple medications. Warfarin, a widely used anticoagulant, interacts with various agents used in palliative care, such as oxycodone, morphine, acetaminophen, and non-steroidal anti-inflammatory drugs (NSAIDs) [1]; thus, an interaction with a concomitant medication is crucially important. However, there are no reports of the interaction of warfarin with methadone. We report a case of a patient receiving warfarin for atrial fibrillation when methadone was introduced for pain control with monitoring of the prothrombin time-international normalized ratio (PT-INR) and deduced the pharmacological background.

## Case presentation

During treatment with axitinib, a 60-year-old male was emergently admitted to our university hospital for the sudden onset of severe back pain. Two years prior, he underwent a right nephrectomy with remaining metastasis to bone, lung, small intestine, and bilateral adrenal glands. He received warfarin 3.5 mg/day for atrial fibrillation and tapentadol 100 mg p.o. daily for pain relief. The PT-INR was maintained at >2.2. His history included diabetes mellitus and hypertension, but his laboratory tests were unremarkable with the exception that his eGFR was 34 ml/min.

He was transferred to the palliative care team for pain control. Initially, a fentanyl dermal patch was used instead of tapentadol to avoid interactions with warfarin. Abdominal CT imaging revealed the vertebral body of the ninth thoracic vertebra was occupied by bone metastasis and crushed, which caused his back pain.

**Fig. 1:**
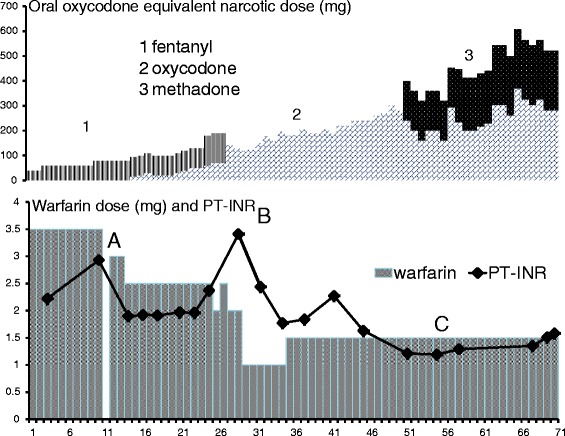
Transition of oral oxycodone equivalent narcotic dose, warfarin dose, and PT-INR. *Horizontal axis*: number of days elapsed since hospitalization (days). *Figure top*: transition of oral oxycodone equivalent narcotic dose. *Vertical axis*: oral oxycodone equivalent narcotic dose (mg). *Figure bottom*: transition of warfarin dose and PT-INR. *Vertical axis*: warfarin dose (mg) and PT-INR. **a** The peak of PT-INR after administration of acetaminophen. **b** The peak of PT-INR after administration of oxycodone. **c** The plateau of PT-INR after administration of methadone. Methadone was not converted to form a ratio with other opioids; the graph was prepared by converting methadone 15 mg to oral oxycodone 80 mg [13]

Decreased renal function prevented the use of NSAIDs and new oral anticoagulants instead of warfarin. We started concomitant administration of oxycodone and acetaminophen under monitoring of the PT-INR because acetaminophen increased the PT-INR to 2.93 (Fig. 1).

On day 15, a continuous intravenous infusion of oxycodone was introduced, in increments of the dose, resulting in the increase of the PT-INR to 3.41, which is required to reduce the dose of warfarin to 1.5 mg. Because of a lack of effective pain relief with oxycodone, acetaminophen, and additional adjuvant analgesics, methadone was introduced on day 52 and the dose was gradually increased. The PT-INR was not changed and the dose of warfarin was not changed. An infusion of oxycodone (130 mg/day) and oral methadone (45 mg/day) was used to allow the patient to walk in his room, and he was later transferred to the palliative hospital.

### Discussion

Drug interactions can occur in any process of absorption, distribution, excretion, and metabolism; the latter is the most frequent [2]. Drug metabolism is classified into the first and the second phase reactions and the first phase oxidation by cytochrome P450 (CYP) is particularly important, and there is a large number of CYP isoforms with substrate specificity. Fentanyl, oxycodone, and methadone used in our case are substrates of CYPs. Although CYP3A4 and CYP2D6 are primarily responsible for opioid metabolism, CYP1A2, 2B6, 2C8, 2C9, and 2C19 are also involved in the metabolism of methadone [3]. Competitive metabolic inhibition can occur when the concomitantly administered drugs are metabolized by the same CYP isoforms [3]. However, the metabolism is complicated because a drug may inhibit the metabolic activity of CYP isoforms other than those involved in its own metabolism as shown by quinidine, a substrate of CYP3A4 that strongly inhibits CYP2D6 activity [4]. Besides the CYP isoforms involved in the metabolism of each drug, the drug-drug interaction should also be examined.

Although acetaminophen and warfarin are metabolized by different CYP isoforms (CYP2E1 and 2C9, respectively) [5], acetaminophen administered ≥ 2 g/day for ≥3 consecutive days extend PT-INR via an inhibition of the metabolism of warfarin, which might be result from the degradation of vitamin K cycle due to depletion of glutathione induced by acetaminophen [5]. Oxycodone is metabolized to noroxycodone by CYP3A4 and to a lesser extent, to oxymorphone by CYP2D6. Concomitant use of CYP3A4 inhibitors results in increased effects of oxycodone [3]. Hosokawa et al. [6] showed the enhanced anticoagulant effect of warfarin after co-administration with oxycodone 10 mg in seven of nine patients, although the mechanism is not known. In this case, the induction of acetaminophen and oxycodone required a reduction in the dose of warfarin. On the other hand, we did not need to adjust the dose of warfarin after introducing and increasing the dose of methadone.

Methadone has a complex pharmacokinetic profile marked by substantial interindividual pharmacokinetic variability and is associated with numerous drug interactions [3]. The clearance of methadone is increased or decreased by CYP3A4 inducers and inhibitors [7, 8], which may also be influenced by CYP2B6 inducers and inhibitors [9], as shown by an increased plasma concentration of methadone by paroxetine [10]. Because warfarin as well as methadone is metabolized by CYP2C9, it is not surprising that methadone extends the effect of warfarin; however, their interaction caused by competitive inhibition of CYP2C9 is unknown so far [3].

Crettol et al. [11] attempted to associate CYP2B6, CYP2C9, and CYP2C19 polymorphisms with methadone plasma levels in 209 patients during methadone maintenance but found positive associations for only the CYP2B6 variants. Methadone is metabolized by various CYPs; the most important are CYP3A4, CYP2D6, and CYP2B6. The relative importance of CYP2C9 is low.

Not only is methadone metabolized by CYP3A4, but it also induces CYP3A4 [12], which can metabolize co-administered oxycodone. In this case, we partially changed the opioid from oxycodone to methadone to increase the concentration of narcotics. Under the condition of this clinically common case, we could not find any interaction between warfarin and methadone.

## Conclusions

We report a case of methadone introduced to a patient administered with warfarin that resulted in no change to the PT-INR during this period. When warfarin and methadone are used together, we have to consider their interaction by comparing the competitive inhibition of CYP2C9 to the induction of CYP3A4 by methadone, because CYP3A4 metabolize various drugs including oxycodone. The pharmacokinetics of methadone varies greatly from person to person; thus, physical observation and monitoring of PT-INR are necessary for drug co-administration.

## Abbreviations

NSAIDs: Non-steroidal anti-inflammatory drugs
PT-INR: Prothrombin time-international normalized ratio
